# Intervention to reduce excessive alcohol consumption and improve comorbidity outcomes in hypertensive or depressed primary care patients: two parallel cluster randomized feasibility trials

**DOI:** 10.1186/1745-6215-15-235

**Published:** 2014-06-19

**Authors:** Graeme B Wilson, Catherine Wray, Ruth McGovern, Dorothy Newbury-Birch, Elaine McColl, Ann Crosland, Chris Speed, Paul Cassidy, Dave Tomson, Shona Haining, Denise Howel, Eileen FS Kaner

**Affiliations:** 1 Institute of Health and Society, Newcastle University, Baddiley Clark Building, Richardson Road, Newcastle upon Tyne NE2 4AX, UK; 2 Department of Pharmacy, Health and Well-being, Sunderland University, Chester Road, Sunderland SR1 3SD, UK; 3 Teams Medical Practice, Watson Street, Gateshead NE8 2PQ, UK; 4 Collingwood Health Group, Brookland Terrace, North Tyneside NE29 8EA, UK; 5 NHS North of England Commissioning Support Unit, Goldcrest Way, Newcastle upon Tyne NE15 8NY, UK

**Keywords:** Alcohol, Screening, Brief intervention, Comorbid, Hypertension, Depression, Primary care, Trial, Preventive, Feasibility

## Abstract

**Background:**

Many primary care patients with raised blood pressure or depression drink potentially hazardous levels of alcohol. Brief interventions (BI) to reduce alcohol consumption may improve comorbid conditions and reduce the risk of future alcohol problems. However, research has not established their effectiveness in this patient population. This study aimed to establish the feasibility of definitive trials of BI to reduce excessive drinking in primary care patients with hypertension or mild to moderate depression.

**Methods:**

Thirteen general practices in North East England were randomized to the intervention or control arm of one of two parallel pilot trials. Adult patients drinking excessively and diagnosed with hypertension or mild-to-moderate depression received the Alcohol Use Disorders Identification Test (AUDIT) by postal survey. Consenting respondents scoring more than 7 on AUDIT (score range 0 to 40) received brief alcohol consumption advice plus an information leaflet (intervention) or an information leaflet alone (control) with follow-up at six months. Measurements included the numbers of patients eligible, recruited, and retained, and the AUDIT score and systolic/diastolic blood pressure of each patient or the nine-item Patient Health Questionnaire (PHQ-9) score. Acceptability was assessed via practitioner feedback and patient willingness to be screened, recruited, and retained at follow-up.

**Results:**

In the hypertension trial, 1709 of 33,813 adult patients (5.1%) were eligible and were surveyed. Among the eligible patients, 468 (27.4%) returned questionnaires; 166 (9.6% of those surveyed) screened positively on AUDIT and 83 (4.8% of those surveyed) were recruited (50.0% of positive screens). Sixty-seven cases (80.7% of recruited patients) completed follow-up at six months. In the depression trial, 1,044 of 73,146 adult patients (1.4%) were eligible and surveyed. Among these eligible patients, 215 (20.6%) responded; 104 (10.0% of those surveyed) screened positively on AUDIT and 29 (2.8% of those surveyed) were recruited (27.9% of positive screens). Nineteen cases (65.5% of recruited patients) completed follow-up at six months.

**Conclusions:**

Recruitment and retention rates were higher in the hypertension trial than in the depression trial. A full brief intervention trial appears feasible for primary care patients with hypertension who drink excessively. High AUDIT scores in the depression trial suggest the importance of alcohol intervention in this group. However, future work may require alternative screening and measurement procedures.

**Trial registration:**

Current Controlled Trials ISRCTN89156543; registered 21 October 2013.

## Background

Excessive alcohol consumption is currently the second greatest risk to public health in developed countries [1] and it affects a wide range of physical and mental health problems [2,3], including hypertension and depression. Hypertension is the leading risk factor for global disease burden [4] with 50% prevalence in those aged over 50 years in the United Kingdom [5]. Depression contributes 12% of the total burden of non-fatal global disease and is likely to be ranked as the world’s second most disabling disease after cardiovascular disease by 2020 [6]. In the United Kingdom, mixed anxiety and depression is prevalent in 11% of individuals aged between 16 and 74 years [7]. Alcohol consumption also has one of the strongest associations with adverse blood pressure changes [8-13] and excessive drinking is a risk factor for developing hypertension [14], strokes [15], and cardiovascular disease [11,16,17], while the association between depression and alcohol use is well-established, with high rates of co-occurrence recognized [11,18-20].

Addressing the co-occurrence of excessive drinking and these comorbid conditions should be a key priority for public health. A recent five-year cohort study provided support for excessive drinking as a cause of depressive symptoms [21] and reductions in alcohol consumption can lead to reductions in blood pressure [8,10,12,22-24]. Comorbidity may lead to worse alcohol-related outcomes than an alcohol use disorder alone [19,25]; however, comorbid populations may be particularly responsive to interventions to reduce their drinking [26,27]. Primary care has considerable potential as a setting for such interventions. Excessive drinkers make up 20% of patients presenting to primary care, and hypertension and depression are two of their most common comorbid conditions; patients with chronic conditions are also regular and frequent users of primary care [28]. A 2007 audit in 34 primary care practices in one United Kingdom regional authority found that of 30,911 patients on a hypertension register and 28,697 patients on a depression register, 26% were excessive drinkers [29]. Until recently, most practitioners under-recognized the role of excessive drinking in the chronic conditions that they treated [30].

Screening to facilitate case recognition with brief alcohol intervention, consisting of structured advice and/or behaviour change counselling (SBI), is a strategy that can reduce excessive drinking in primary care patients by approximately seven drinks each week [31]. Such a reduction in alcohol consumption could have an impact on comorbid physical or mental health problems. However, most previous trials of brief intervention to reduce excessive drinking have excluded patients diagnosed with specific comorbidities and have not measured the impact of reduced drinking on those conditions [32]. A recent systematic review [33] identified only 11 brief intervention trials where participants had a recognized substance use problem (alcohol, tobacco, or other drug use) as well as a comorbid physical condition (3 trials) or mental health problems (8 trials). This review found positive outcomes of brief intervention in three trials where substance-using patients had a comorbidity of hypertension [26,34] or another physical condition [35]; both of the former trials were based in primary care. The impact of brief intervention in substance-using patients with comorbid mental health problems was equivocal, with both positive and null effects [33]. The two trials that focused on alcohol use reported positive drinking outcomes in hospital patients with schizophrenia and a range of psychiatric conditions [36,37]. However, these two studies included patients with relatively severe mental health problems and found no changes in mental health states. The findings of this review suggest that the evidence from relevant trials in this field is insufficient to draw firm conclusions about the impact of brief alcohol intervention on linked health conditions, particularly in a primary care context.

If feasible, a trial of SBI for alcohol in patients with comorbid health conditions that measured changes in health state as well as in drinking would address this gap in knowledge. In line with recommendations for the development of complex interventions [38] the current pilot trials aimed to establish whether a definitive randomized controlled trial of brief alcohol intervention in patients drinking excessively with a comorbidity of hypertension or depression would be feasible in the United Kingdom. Specifically, the objectives of this study were to establish: rates of eligibility, recruitment, and retention in the pilot trials in the two patient populations (as the feasibility objective, these rates were the primary focus for the pilot); numbers of primary care patients identifiable with hypertension or mild/moderate depression who were excessive drinkers (scoring 8 or above on the AUDIT tool) [39]; acceptability to patients and practitioners of intervention and research materials and procedures; practicality of outcome measures of health variables (alcohol use disorder risk, blood pressure, and depression) that were proposed for a full trial, operationalized as the number of patients in which these measures were achieved.

This article reports the design and results of these parallel trials, along with a discussion of these in relation to published literature and considers whether full trials are supported by this feasibility work.

## Methods

### Design

Two parallel two-arm cluster randomized controlled pilot trials were carried out. On advice from our trial steering group, we focused on only one comorbid condition per practice to reduce the burden of research work on staff. General practices were each randomly allocated to one trial using computer-generated random numbers (defined by comorbidity conditions of either hypertension or mild to moderate depression) and then one arm of that trial (either brief intervention (structured brief advice) or control (information leaflet)). Cluster randomization was chosen to match the likely design in a definitive trial to prevent contamination from GPs trained to deliver brief alcohol advice using these skills with control patients. Randomization to trials was undertaken to avoid giving practices the opportunity to select a trial corresponding to a preferred condition, which might introduce bias. Patients were not informed which arm of the trial their practice had been allocated to [40].

### Setting

Eighty-two general practices in the North East of England were invited to take part in the study in two waves. In the first wave, 27 general practices with prior involvement in research (‘research practices’) from three primary care trusts (PCTs) were approached, as well as all remaining practices (n = 24) with a patient list over 4,000 in one of those PCTs. Fourteen of the research practices were approached by an appointed research facilitator working for one PCT and the remainder received an email invitation from the university-based research team addressed to either the practice managers or one of the GP partners, with telephone follow-ups on the practice managers after two weeks. In a second wave of recruitment eight months later, the research team re-invited eight practices that had been too busy at the first wave (one a research practice) plus five practices with prior involvement in research from three neighboring PCTs, and 26 further practices with a patient list over 9,000 from across all six PCTs.

Prior to randomization, all practices who agreed to participate were asked to complete a series of anonymous searches of their databases, respectively for male and female patients aged 18 or above who had a coded record of: drinking excessively (weekly alcohol consumption recorded as more than 21 units per week for men or more than 14 units per week for women, or scoring positively on a validated screening tool for alcohol consumption such as AUDIT, FAST, or SASQ); a diagnosis of hypertension; depression or low mood but no referral to specialist services for this; both excessive drinking and a diagnosis of hypertension; both excessive drinking and depression (or low mood).

The database search strategy is provided in detail in Additional file 1. No date limit was set on the searches, apart from those conducted at the last five practices recruited. As screening and recruitment proceeded it had become apparent that many patients were excluded at baseline appointments due to their symptoms having improved since their record of depression on the database. To reduce the burden upon patients of attending an appointment only to be excluded from the study, the depression codes in searches for eligible patients at the last five practices were therefore limited to the preceding year. Those drinking excessively and having a recorded diagnosis of hypertension, or drinking excessively and having mild to moderate depression, were deemed to meet initial eligibility criteria (referred to henceforth as ‘database eligible’).

The eight practices (median adult patient list size 7917, range 5562 to 13611) that had identified the largest numbers of comorbid patients were approached to participate in the two pilot trials; they included four research practices. These practices were randomized equally to the two arms in each of the two parallel trials, that is, two practices in each of the depression control (DC), depression intervention (DI), hypertension control (HC), and hypertension intervention (HI) categories. Before questionnaires were sent out to patients one practice (allocated to DI) withdrew because there was no funding to support their involvement and another (allocated to HI) withdrew because of GPs’ subsequent concerns that patients might not wish to be asked about their alcohol consumption. One of these practices agreed that the results of their database searches could still be included to establish prevalence, the other practice did not respond when this was queried. A ninth research practice that had also identified a large number of comorbid patients was approached, replacing the practice which had dropped out of the depression trial (the first to withdraw). At the second wave, a recruitment target of 30 patients per arm had already been exceeded in the hypertension trial; practices approached were therefore invited to participate in the depression trial only. Six practices (median adult patient list size 4848, range 4694 to 10464), including one research practice, agreed and were randomized in equal numbers to either DI or DC. The final number of practices per trial arm was therefore two practices in HC, one practice in HI, five practices in DC, and five practices in DI.

### Participants

Patients meeting initial eligibility criteria but confirmed by GPs as having a severe mental health disorder, cognitive impairment, or terminal illness, or accessing treatment for alcohol use, were excluded from further consideration. All remaining ‘database eligible’ patients were sent a trial pack containing a letter on practice-headed paper with a GP’s signature inviting them to participate by returning a questionnaire, a patient information leaflet relevant to the trial to which their practice was allocated, a screening questionnaire comprising the 10 items of the Alcohol Use Disorder Identification Tool (AUDIT) tool [39] and requesting demographic and contact details for patients who wished to be considered for the trial, and a reply-paid envelope addressed to Newcastle University for questionnaire return. No subsequent reminders were sent.

Patients who returned a questionnaire with a score of 8 or more on the AUDIT and who volunteered their contact details were deemed ‘screen eligible’. A member of the research team contacted these participants directly by telephone to arrange an appointment with a researcher at the patient’s surgery (the premises at which they were registered with their GP practice); appointments were not offered in any other location. If the participant did not attend the appointment a further appointment was negotiated with the patient at a convenient time. Patients were offered a maximum of three separate appointments. Up to seven attempts were made to contact an eligible respondent by phone. If it had not been possible to arrange an appointment after seven phone calls at different times of day (including weekends and evenings) a final letter was sent offering an appointment at a given time; no further appointments were offered if the participant did not attend on this occasion. If at any point a respondent indicated a wish to withdraw from the study, they were thanked for their interest and not contacted any further. Any data collected on the patient to that point was retained and participants were not asked their reasons for withdrawing if they chose to do so, in keeping with the terms of the consent agreement.

At the appointment, the trial was explained in more depth and any queries discussed, following which the patient was invited to give written consent to participate. A baseline measurement of participants’ comorbid condition was taken as described under ‘Measures’ below. If the participant was still eligible at this stage (‘fully eligible’), the intervention was delivered by the researcher according to allocation status. Recruitment commenced on 31 January 2011, with the final follow-up interview carried out on 28 February 2012.

### Interventions

At all baseline interviews patients received either an advice leaflet produced by the British Heart Foundation (hypertension trial) or a leaflet on depression produced by a regional NHS organization (depression trial). In control practices this was the only deviation from usual care following baseline assessment.

At practices randomized to the intervention arms only, university research staff (GW and CW) delivered five minutes of structured advice on alcohol consumption at baseline, tailored to the patient’s physical or mental health comorbidity. The brief advice (see Additional file 2) consisted of personalized, structured feedback to patients about their level of alcohol-related risk or harm, a visual normative comparison of where their drinking behaviour sat in relation to population norms, health benefits associated with reducing alcohol consumption from their current levels, and practical suggestions on how to reduce drinking levels.

Materials were designed for the study and based on those used in previous research on brief interventions (BI) in primary care [41]. Research staff received training in intervention delivery from an experienced alcohol interventionist (RM) who also observed the initial four interventions in the depression trial in order to assess fidelity. On these occasions the observer’s presence and the voluntary nature of observation was explained beforehand and patients were invited to ask any questions and give verbal consent before observing.

### Outcomes

#### ***Primary outcome for a main study***

It was envisaged that the primary outcome in a main study following either trial would be risk of alcohol use disorders. This was to be assessed using AUDIT, which is considered the ‘gold standard’ screening tool for identifying excessive drinking and is brief, reliable, and valid for use in primary care settings [39]. Ten items are each rated from 0 to 4 (full score range 0 to 40); total scores of 8 to 15 (inclusive) identify hazardous alcohol use whilst total scores of 16 to 19 (inclusive) identify harmful alcohol use, and a score of more than or equal to 20 indicates probable dependence [3]. A cutoff score of 8 was adopted in both trials, at which point AUDIT has a sensitivity of 92% and a specificity of 94% for risk drinking in adults [39].

#### ***Secondary outcomes for a main study***

The secondary outcomes envisaged for a main trial based on the hypertension trial were systolic and diastolic blood pressure. These were measured in the pilot hypertension trial by a researcher taking single, seated readings of systolic and diastolic blood pressure in the patient’s surgery at baseline and follow-up. A commercially available blood pressure monitor with arm cuff (Kinetik Medical Devices Ltd., Elstree, United Kingdom; endorsed by the British Hypertension Society) was used in accordance with the manufacturer’s instructions. At baseline this constituted measurement of outcome rather than a clinical assessment; no eligibility criterion was applied at this stage of the hypertension trial.

The secondary outcome envisaged for a main trial based on the depression trial was severity of depression, measured at baseline and six-month follow-up using the nine-item Patient Health Questionnaire (PHQ-9). This is a brief, reliable, and valid screening tool that has been extensively used for clinical and research purposes with primary care patients [42,43] and is a responsive measure of outcome in depression treatment [44]. Nine items are each rated from 0 to 3 (full score range 0 to 27). The PHQ-9 has demonstrated an 88% sensitivity and an 88% specificity for detecting mood disorders in adult primary care patients using a cutoff score of 10 or more [45]. Consenting patients in the depression trial were included as eligible at the baseline appointment if they scored 5 to 19 inclusive, which indicates mild as well as moderate and moderately severe depression [45,46]. Patients scoring outside this range were excluded: a score below 5 indicated a healthy range, and a score over 19 indicated a patient suffering severe mental health problems, for whom research participation might represent an unacceptable burden.

Pilot studies are a valuable means to assess practicality of primary and secondary outcome measures for a main trial [47]. For this purpose, descriptive statistics were calculated for all the measures described above (AUDIT, systolic and diastolic blood pressure, and PHQ-9 scores).

#### ***Feasibility outcomes***

Feasibility was based on a combination of acceptability and efficiency measures. An acceptable identification strategy was defined as the ability to successfully identify from patient notes (database eligible) a pool of hazardous or harmful drinkers with a comorbidity of hypertension or mild to moderate depression who were willing to be screened on these parameters in their general practice. Efficient recruitment and retention was defined as the ability to recruit eligible (screen positive) patients into the intervention stage of the study and retain them at six-month follow-up. The measures of these feasibility outcomes were the rates for each pilot trial of patient eligibility; recruitment; and retention at six-month follow-up.

#### ***Follow-up***

Six months after the baseline appointment, all participants were contacted by a researcher via telephone and asked to participate in a follow-up appointment at which outcome measurements were repeated (AUDIT and PHQ-9 score or blood pressure reading). This was arranged at a convenient time for them at their GP practice. If it did not prove possible to contact a participant by phone, a letter was sent from the research team inviting them to an appointment at their GP surgery at a fixed time. Any views expressed by patients at these appointments were noted.

#### ***Sample size***

A sample size calculation was not carried out to determine the number of clusters or patients for either of these trials; sample size calculations are not a prerequisite for pilot studies [48] and general recommendations are not available to determine numbers of clusters if randomizing by cluster in these studies [49]. Following recommendations for pilot trials randomizing by individual patients (also cited for trials randomizing by cluster) [40,49], a target figure of 30 patients recruited per arm was sought at baseline (120 patients overall).

#### ***Feasibility criteria***

##### 

**Acceptability of identification strategy** Previous screening in primary care has found that 35% of adult patients are likely to be hazardous or harmful drinkers [50]. However, such studies generally focus on alcohol consumption alone. The size of the comorbid patient group is not known. We did not set a specific figure for the number of patients we needed to identify as database eligible. The key issues were to establish if Read code data (the standard clinical terminology system used in General Practice in the United Kingdom) were sufficient to identify cases and if the procedure was acceptable to practice staff and patients. Acceptability to patients was judged by their response to the invitation to attend for screening and informed by feedback from practitioners and patients in the course of the study.

##### 

**Efficient recruitment and retention** Recent work in the United Kingdom found that approximately 25% of screened adult male patients in primary care who scored more than 7 on AUDIT were willing to be recruited into an alcohol intervention trial [51] and published recommendations suggest that a retention rate of more than 80% at follow-up would support feasibility [52]. Thus recruitment of 25% of screened patients who scored more than 7 on AUDIT and a retention rate of 80% at the six-month follow-up were taken as reasonable success criteria for a definitive trial.

### Analysis

Eligibility, recruitment, and retention rates were calculated as percentages for the two pilot trials. Descriptive statistics (mean, standard deviation, and percentages as appropriate) were used to characterize trial participants and summarize trial outcomes by arm. Rates of eligibility, recruitment, and retention between trials were compared using chi-square tests, and AUDIT scores and characteristics of consenters and non-consenters at baseline were compared using *t*-tests.

A sample size calculation for a future trial was carried out based on estimates of rates of eligibility, recruitment, retention, and response from this feasibility study, and publicly available information on average practice size in England. Since it is unlikely that intraclass correlation coefficients can be estimated with any degree of precision from a pilot trial [49] and since there were few clusters in this feasibility study, an estimate was based instead on other studies in general practice.

## Ethical approval

The study received ethical approval from the County Durham and Tees Valley NHS Research Ethics Committee (Reference 10/H0908/35, 21 May 2010). Governance approval for the research was gained from the PCTs acting as trial sites.

## Results

### Recruiting practices

Of the 82 practices approached, 29 (35.4%) agreed to participate; 5 of these practices agreed only to the search of their database, without subsequent trial participation. Overall, 51.5% of research practices agreed (including 15.2% that agreed only to the database search) compared with 24.4% of non-research practices (including 2.0% agreeing only to the database search). The median patient list size for participating practices was 9,011 (range 3,000 to 29,778) compared with 10,009 (range 810 to 14,847) for non-participating practices. The median patient list size for practices agreeing only to the database search was 12,625 (range 5,513 to 29,778). Of the 26 practices across both waves of recruitment that gave a reason for declining, 30.8% indicated that they were too busy, and 46.2% stated that the GPs were not interested.

Staff with access rights to identifiable data at each of the 29 practices were asked to complete the search strategy. Three practices returned partial information only: one practice manager terminated the searches as she felt they required too much of the staff member’s time, another considered that GPs’ extensive use of free text coding meant that some search results would not be valid, and another did not respond to requests to complete the searches. A further practice withdrew from the study because funds were not available to cover practice costs for recruitment and did not respond to a query as to whether they were happy to contribute prevalence data to the study. Twenty-five practices (86.2% of those agreeing to participate, 30.5% of all approached) with a median adult patient list of 7,328 (range 3,490 to 25,903) were able to complete and provide anonymous searches of their databases. Fourteen of these practices (56.0%) were research practices.

### Prevalence

**Figure 1:**
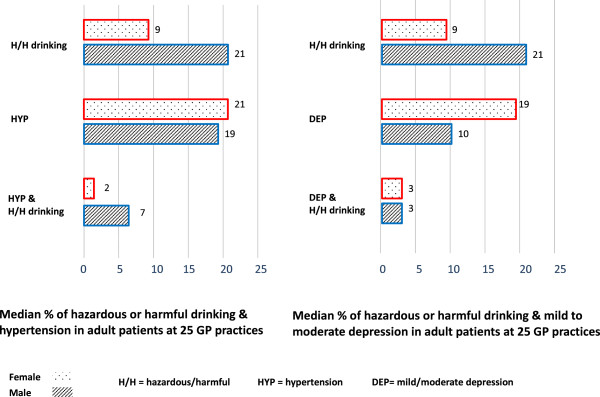
**Prevalence at 25 practices of hazardous or harmful drinking, hypertension, mild/moderate depression, and comorbidities.** Dotted bars indicate female, lined bars indicate male. DEP, depression, H/H, hazardous/harmful drinking, HYP, hypertension.

Prevalence estimates were derived from completed searches at 25 general practices with adult patient lists totaling 203,691. Of these patients, 67.4% had a record of weekly alcohol units consumed, and 13.2% of all adult patients had a record of exceeding low risk drinking guidelines of 21/14 (m/f) units of alcohol per week, with twice as many men as women recorded as drinking to excess (Figure 1). Of the adult patients recorded, 19.1% as hypertensive; 4.0% of adult patients were both hypertensive and drank excessively. Of the adult patients recorded, 14.3% had a record of mild or moderate depression or low mood; 2.7% of adult patients had this record and also drank excessively. A slightly greater proportion of female patients than male patients were recorded as having experienced each comorbid condition (Figure 1). At the five practices which ran a search limited to the previous year for depression terms, the search indicated comorbidity for only 1% of adult patients.

### Recruitment and retention of patients

**Figure 2:**
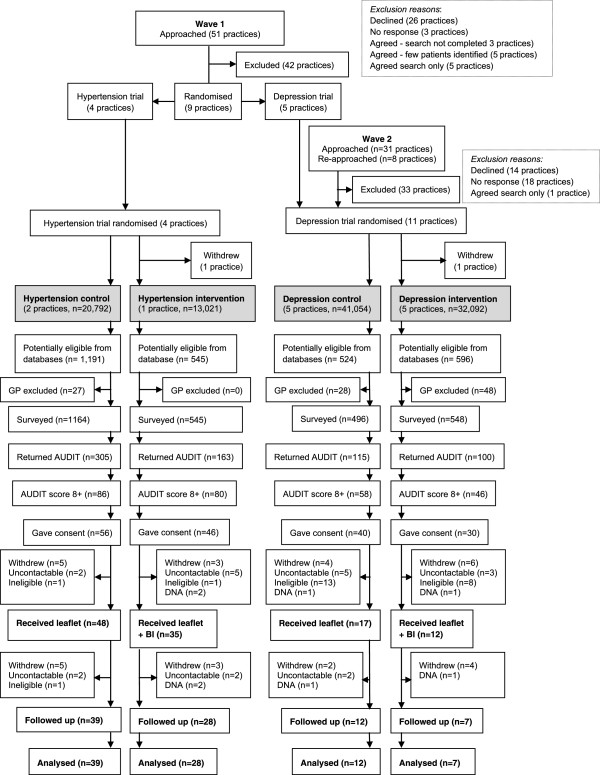
**CONSORT flow diagram for the Comorbidities and Brief Interventions in Northeast England study (ComBIne).** BI, brief interventions; DNA, did not attend.

Patients were recruited to the study between January and August 2011 and the final follow-up appointment took place in February 2012. Figure 2 shows the CONSORT diagram of the flow of participants through the trials. Practices in the hypertension arm were fewer and larger, with the three GP practices in the hypertension trial having lists of 7,181, 13,611, and 13,021 adult patients respectively, while the ten practices in the depression trial had a median adult patient list of 7,656 (range 4,694 to 10,464).

### Hypertension pilot trial

In the hypertension trial, 1,736 patients (73.4% male) were ‘database eligible’, having a record of excessive drinking and raised blood pressure. GPs excluded 27 of these patients (1.5%) and the remaining 1,709 received a questionnaire by post. Out of those surveyed, 468 returned a questionnaire (27.4% of the 1,709 surveyed), including four who did not answer the AUDIT questions. Of these 468 respondents, 166 (35.5% of respondents, 9.6% of those ‘database eligible’) scored positively on AUDIT. Of these respondents, 102 were ‘screen eligible’ in that they also gave consent to be contacted for interview (5.9% of ‘database eligible’, 21.8% of questionnaire respondents, 61.4% of those scoring positively on the AUDIT). Eighty three individuals were recruited to the trial (4.8% of ‘database eligible’, 81.4% of ‘screen eligible’). Sixty seven participants were retained at six months (3.8% of ‘database eligible’, 65.7% of ‘screen eligible’, 80.7% of those recruited), with little difference in retention rates between the control arm (81.2%) and the intervention arm (80.0%).

Of the 19 ‘screen eligible’ patients who were not recruited, seven could not be contacted after an average of seven attempts, eight withdrew consent when contacted, two repeatedly failed to attend appointments, and two reported at their appointment that they were currently receiving treatment for alcohol problems and therefore were not deemed fully eligible. At six months, four individuals could not be contacted, three did not attend appointments, and nine participants withdrew consent (one following an appointment missed by the researcher).

### Depression pilot trial

In the depression trial, 1,120 patients (50.3% male) were ‘database eligible’ and GPs excluded 76 of these patients (6.8%). Two hundred and fifteen patients returned a questionnaire (20.6% of the 1044 surveyed), including one who did not answer the AUDIT, 104 (48.4% of respondents, 9.3% of those ‘database eligible’) scored positively on AUDIT. Seventy of these respondents gave consent to be contacted for interview and were deemed ‘screen eligible’ (6.3% of ‘database eligible’ 32.6% of questionnaire respondents, 67.3% of those scoring positive on the AUDIT). Twenty-nine individuals (2.6% of ‘database eligible’, 41.4% of ‘screen eligible’) were recruited to the trial. Nineteen participants (1.7% of ‘database eligible’, 27.1% of ‘screen eligible’, 65.5% of those recruited) were retained at six months, and the difference between retention rates of those recruited to the control arm (70.6%) and the intervention arm (58.3%) was greater than in the hypertension trial, but not statistically significant.

Of the 41 ‘screen eligible’ patients who were not recruited, 21 were not deemed fully eligible: one scored higher than 19 and 17 scored lower than 5 on PHQ-9 when measured at the baseline appointment, two patients indicated they were receiving treatment for alcohol problems, and a further patient was found to suffer cognitive impairment. In addition, eight ‘screen eligible’ patients could not be contacted after an average of seven attempts, ten withdrew consent when contacted, and two repeatedly failed to attend appointments. At six months, two participants could not be contacted, two repeatedly failed to attend appointments and six patients withdrew consent (one following an appointment missed by the researcher).

There were significant trends towards higher rates of questionnaire return and of patient recruitment among baseline appointment attendees in the hypertension than in the depression trial (*x*^2^ = 16.02, *P* <0.001 in respect of questionnaire return; *x*^2^ = 29.16, *P* <0.001 in respect of patient recruitment), and towards a higher rate of questionnaire respondents screening positive (AUDIT score >7) in the depression than in the hypertension trial (*x*^2^ = 10.26, *P* <0.01). There were no significant trends between the trials in proportions of respondents who were ‘screen eligible’, baseline appointments attended, or recruited participants who were retained at six months.

### Characteristics at baseline

AUDIT scores for survey respondents in both trials (n = 678), and characteristics for those who answered the demographic questions (n = 381 to n = 678) are reported in Table 1, and were compared to consider any possible selection bias in the samples [47]. Respondents supplying demographic information in the depression trial were younger and more likely to be employed or female than those in the hypertension trial. Comparing responses for those who returned questionnaires in each trial, the mean AUDIT score was greater among depression trial respondents than among hypertension trial respondents, with the mean AUDIT score of respondents in the depression trial indicating hazardous drinking. In the hypertension trial, there was little difference in age, sex, or proportion in employment between survey respondents supplying this information in the intervention and control arms, however, mean AUDIT scores and the proportion scoring positively on AUDIT were both higher in the intervention arm (mean AUDIT score 7.9; SD 4.7; 49.1% positive) than in the control arm (mean AUDIT score 6.5; SD 4.7; 28.6% positive). In the depression trial, all respondent characteristics at baseline were similar across intervention and control arms.

**Table 1:** Demographic characteristics (age, sex, and employment rates) and AUDIT scores of survey respondents in each trial

	**Hypertension**	**Depression**
**Mean age in years (SD)**	65 (10.6)	54 (14.5)
^***1***^n_h_ = 266, n_d_ = 137		
**% male**	83.3	59.7
n_h_ = 275, n_d_ = 139		
**% not in paid employment**	72.8	49.2
n_h_ = 256, n_d_ = 125		
**Mean AUDIT score (SD)**	7.0 (4.8)	10 (8.4)
n_h_ = 464, n_d_ = 214		
**% AUDIT positive**	35.8	49.1
n_h_ = 464, n_d_=,214		

^**1**^n_h_ and n_d_ give the total number in each trial supplying relevant data (hypertension and depression, respectively).

AUDIT scores were also compared in each trial between all those who gave consent to being contacted and all those who did not, and between AUDIT positive respondents who gave consent and AUDIT positive respondents who did not. In the hypertension trial, AUDIT scores were significantly higher for all respondents who gave consent to contact (n = 249; mean 7.5; SD 5.25) compared to those who did not (n = 215; mean 6.4; SD 4.10) (1.13 mean difference; 95% CI 0.26 to 2.00; *P* = 0.011). Among the 166 respondents scoring positively on AUDIT, AUDIT scores were not significantly different between those who gave consent to contact and those who did not. In the depression trial, AUDIT scores were significantly higher for all respondents who gave consent (n = 124; mean 11.9; SD 9.52) compared to those who did not (n = 87; mean 7.7; SD 5.75) (4.256 mean difference; 95% CI 2.01 to 6.51; *P* <0.001). Among the 104 respondents scoring positively on AUDIT, AUDIT scores were significantly higher among those who gave consent to contact (mean 18.1; SD 8.51) than among those who did not (mean 13.0; SD 5.62) (5.087 mean difference; 95% CI 1.93 to 8.25; *P* = 0.002).

Mean AUDIT scores and demographic characteristics at baseline are reported in Table 2 for those who consented to being contacted by a researcher and were subsequently recruited to either of the two trials at baseline appointments. Among ‘screen eligible’ respondents who gave consent to contact in the hypertension trial, those recruited were older (mean 64 years, SD 9.19) with a lower mean AUDIT score (mean 12, SD 4.69) than those who were not recruited (mean 55 years, SD 11.73 and mean score 13, SD 7.40), and were more likely to be male (88.0% compared with 75.0%). Among screen eligible respondents giving consent to contact in the depression trial, those recruited were somewhat older (mean 53 years; SD 14.78) with a lower mean AUDIT score (mean 17; SD 8.29) than those who were not recruited (mean 48 years, SD 14.71; mean score 21, SD 8.37), and were more likely to be male (67.4% compared with 59.3%). None of these differences were statistically significant.

**Table 2:** Demographic characteristics and outcome measures at baseline and at six months for patients recruited to the trial

	**Hypertension**		**Depression**	
	**Control**	**Intervention**	**Control**	**Intervention**
**Mean age in years (SD)**	63 (8.1)	66 (10.4)	53 (13.3)	50 (16.2)
^***1***^n_h_ = 81, n_d_ = 29				
**% male**	89.6	85.7	64.7	75.0
n_h_ = 83, n_d_ = 29				
**% not in paid employment**	77.3	71.4	50.0	72.7
n_h_ = 77, n_d_ = 27				
**BASELINE:**				
**Mean AUDIT (SD)**	12 (4.7)	12 (4.7)	15 (6.4)	20 (9.7)
n_h_ = 83, n_d_ = 29				
**Mean PHQ-9 (SD)**	-	-	10 (4.2)	11 (4.7)
n_d_ = 29				
**Mean Systolic BP (SD)**	153 (19.4)	149 (16.1)	-	-
n_h_ = 83				
**Mean Diastolic BP (SD)**	88 (10.1)	87 (8.8)	-	-
n_h_ = 83				
**FOLLOW-UP:**				
**Mean AUDIT (SD)**	10 (4.7)	9 (3.8)	14 (6.6)	18 (7.3)
n_h_ = 67, n_d_ = 19				
**% scoring <7 on AUDIT**	25.6	35.7	8.3	0.0
n_h_ = 67, n_d_ = 19				
**Mean PHQ-9 (SD)**	-	-	9 (5.9)	8 (6.0)
n_d_ = 19				
**Mean Systolic BP (SD)**	147 (16.4)	149 (16.9)	-	-
n_h_ = 67				
**Mean Diastolic BP (SD)**	90 (11.2)	88 (10.7)	-	-
n_h_ = 67				

^**1**^ n_h_ and n_d_ give the total number in each trial supplying relevant data (hypertension and depression, respectively).

### Outcome measures at follow-up

Mean scores for AUDIT, one-off blood pressure readings, and PHQ-9 at six-month follow-up are also reported in Table 2. In the hypertension trial, 35.7% of followed up on participants in the intervention arm and 25.6% of those in the control arm reported AUDIT scores at follow-up that were below the cutoff for hazardous drinking (7 or less). Among participants followed up whose systolic blood pressure had been 140 mmHg or above at baseline, 21.4% of those in the control arm and 17.4% of those in the intervention arm had a reading below 140 mmHg at follow-up. Among participants followed up whose diastolic blood pressure had been above 85 mmHg at baseline, 12.5% of those in the control arm and 10.5% of those in the intervention arm had a reading below 85 mmHg at follow-up.

In the depression trial, 8.3% of participants in the control arm reported AUDIT scores at follow-up that were below the cutoff for hazardous drinking (7 or less), however, none of the seven participants in the intervention arm scored below this cutoff at follow-up. Of participants followed up, 33.3% in the control arm and 42.9% of those in the intervention arm returned a PHQ-9 score below the cutoff of 5 at follow-up.

Mean changes in outcome measures from baseline are reported in Table 3 for those participants who were retained at follow-up. In both arms of the hypertension trial, mean systolic blood pressure was reduced by an amount (≥2 mm Hg) cited in the literature as likely to have a substantive public health impact at a population level [12,53]. Diastolic blood pressure was increased by a similar amount at follow-up in both trial arms. In both arms of the depression trial, mean change in PHQ-9 scores was smaller than the clinically significant change of 5 points for patients receiving treatment for depression cited by Löwe *et al*. [44].

**Table 3:** Changes in continuous outcome measures from baseline to six months for participants retained at follow-up.

	**Hypertension**		**Depression**	
	**Intervention (n = 28)**	**Control (n = 39)**	**Intervention (n = 7)**	**Control (n = 12)**
	**Mean change**	**Mean change**	**Mean change**	**Mean change**
	**T2-T1 (SD)**	**T2-T1 (SD)**	**T2-T1 (SD)**	**T2-T1 (SD)**
**AUDIT score**	-1.8 (2.92)	-1.5 (5.2)	-3.1 (4.9)	-1.5 (5.0)
**Systolic BP**	-2.0 (17.7)	-3.2 (16.8)	-	-
**Diastolic BP**	2.2 (10.62)	1.8 (9.12)	-	-
**PHQ-9 score**	-	-	-2.9 (5.7)	-0.7 (6.1)

### Acceptability of procedures and materials

Field notes of comments from GP practice staff and patients to the researchers suggested that they generally found the research procedures acceptable. Four practices were unable to complete the database searches due to problems accessing the data, for instance, some practice managers reported that they were unable to search for ranges of scores within scales or indicated that data on alcohol consumption or depression had been entered as free text and therefore could not be used as a search criterion. No adverse effects were reported.

In the hypertension trial practices patients often compared research appointments to the ‘Well Man’ or ‘Well Woman’ clinics they attended regularly to discuss blood pressure, and tended to treat the research appointment positively as another opportunity to monitor their health, for instance comparing their blood pressure reading to that at the most recent clinic. Some patients in the depression trial who were not fully eligible at their baseline appointment because of a PHQ-9 score below 5 commented that they experienced considerable fluctuation in depressive symptoms, or suggested that in recently preceding weeks they might well have returned an eligible score.

All participants who were scheduled to receive the interventions did so. Patients receiving the brief advice did not voice any concerns regarding it, though some hypertensive patients commented afterwards that they were often advised by doctors or nurses to drink less, or were skeptical of the link between their alcohol intake and their blood pressure. For instance, one man pointed out that his last reading at the surgery had been low despite his having drunk excessively the night before. In comparison, participants receiving the brief advice in the depression trial appeared more ready to view alcohol intake as related to their symptoms.

### Sample size calculation for a main trial based on the hypertension trial

The average adult practice list size across the three PCTs from Wave 1 was 6,366 but across England in 2011 this was 4928 [54,55]. Based on this latter figure, one would expect to identify 246 patients per practice as ‘database eligible’ for a hypertension trial and to recruit and retain 10 patients per practice (to have a cluster size of 10). The corresponding figures for a depression trial are 74 ‘database eligible’ and 2 recruited and retained. As a guide for a future trial based on the hypertension pilot study, a sample size calculation was carried out. To have an 80% power of detecting a standardized difference of 0.3, deemed to be a small difference [56], would require outcome data on 176 subjects per trial arm. A loss to follow-up rate of 81% from the feasibility study would inflate this to 217 recruited per arm. Estimating an intraclass correlation coefficient of 0.04 from other studies based in general practice and a cluster size of 10 based on the feasibility study and average numbers of adults in GP practices would further inflate the numbers recruited per arm to 300. The feasibility study found that 5.1% of patients were ‘database eligible’ for a hypertension trial and 4.8% of these were recruited into the trial. This implies that 122,563 patients’ records would need to be screened per arm, implying about 25 practices would be needed per arm if the average numbers of patients per practice aged 18 or above were 4,928.

## Discussion

This study gathered evidence regarding the feasibility of a definitive trial of brief intervention to reduce excessive alcohol consumption in primary care patients with comorbidities of hypertension or mild to moderate depression. Of adult primary care patients identified, 5.1% were as ‘database eligible’ for the hypertension trial and 1.5% were ‘database eligible’ for the depression trial. In the hypertension trial, 4.8% of ‘database eligible’ patients were recruited into the trial, and 80.7% of these were retained at six months. In the depression trial, 2.5% of ‘database eligible’ patients were recruited into the trial, and 65.5% of these were retained at six months. Rates of eligibility, recruitment, and retention in the hypertension trial were consistent with results and recommendations in the literature [50-52], while those in the depression trial fell somewhat short of these rates. Research tasks were not perceived as burdensome by patients in either trial.

In keeping with published recommendations [57], the study was intended as a pilot from the outset, with appropriate aims: to assess recruitment potential, test the research process, and to identify any potential issues in data management ahead of a full trial [40]. Strengths of this study include the use of validated instruments to assess eligibility and measure outcomes and a high rate of retention at six-month follow-up in one of the trials (80.7%). The inclusion of 25 practices in the database searches provides a robust indication of wider prevalence rates, though these may reflect regional patterns of alcohol use and health [58]. However, practices were not found to screen patients regularly for alcohol consumption and therefore no date limits were applied to the searches. Information on alcohol consumption may have been out of date in some cases, leading to both false positives and false negatives in establishing database eligibility. The implementation of more rigorous screening in routine primary care would enhance future efforts to establish prevalence. The observed difference between mean baseline AUDIT scores for the control and intervention arms in the hypertension trial probably reflects the small number of large GP practices in this trial, and suggests that the profiles of patients at those three practices were not similar. Any such confounding influence of a small number of large practices in the pilot trial should be mitigated in a full trial where greater numbers of practices would be recruited over a wider area. It is also a limitation that demographic data (such as age and sex) were not available to assess how respondents and non-respondents differed, and that demographic questions were completed by too few AUDIT positive respondents to indicate whether those who gave permission for contact differed from those who did not. A greater rate of response to the demographic questions might have been achieved by providing a more detailed explanation on the questionnaire and an information leaflet of why this information was important and how it would be used.

The differing rates of recruitment and retention between the two linked trials may reflect characteristics of the sample or target populations. In the hypertension trial, patients were more likely to be retired with the time and inclination to visit their GP surgery [59], while depression trial participants were more likely to be of working age and bringing up children and therefore having less opportunity to attend their GP surgery. Furthermore, men may perceive less of a stigma than women around admitting to excessive drinking [60], although they attend GP surgeries less frequently [61]. Since the hypertension trial participants were mostly male and the depression trial participants mostly female (consistent with profiles of patients with those conditions), sex differences may have facilitated recruitment to the hypertension trial. As a relatively widespread physical condition, high blood pressure may have also have less stigma attached to it than a mental health problem such as depression. It is informative that hypertension participants frequently indicated that they were accustomed to visiting the surgery to discuss their hypertension, and tended to treat the research appointment as a similar demonstration of their conscientiousness or proactive monitoring of this condition. Finally, the symptoms of depression may make patients feel less able to attend the surgery and trials of interventions via the internet for depression have recently been conducted for this reason [61]. Recruitment in this trial might have been higher with the offer of research appointments taking place at patients’ homes.

However, some factors might have been expected to favor participation in the depression trial rather than the hypertension trial. Patients with depression might be expected to be readier to accept and act on advice linking their drinking with their health condition if they experience direct impact of alcohol consumption on their mood. Hypertension patients on the other hand do not experience an immediate discernible change in symptoms after excessive drinking and as such they may be more ready to accept lay reasoning favoring the perceived benefits of drinking than brief advice on links between drinking and their comorbid condition [62].

A legitimate aim for a feasibility study is to inform the selection of outcome measures for a full trial [40,63] and the results can inform such a choice. Variability in the single measurements of blood pressure at baseline and follow-up was relatively high [64], which means there may be considerable uncertainty using this data when planning a definitive trial. One-off measurement is now considered inadequate due to the high variability in blood pressure readings and confounding influences such as the white coat effect or recent activity [65,66]. Aggregate measurement over a period of time offers a more robust indication of blood pressure. Since the study data were gathered, national guidance has been updated to recommend 24-hour ambulatory monitoring or an average reading of home measurements over a week to confirm a clinical diagnosis of hypertension [67]. At the time of the study, these more precise methods were not in common use in primary care. They were liable to constitute an additional intervention if used to measure outcomes because they require patients to focus their attention on their blood pressure over a longer period. However, since this trial was conducted, 24-hour blood pressure measurement has been added to government recommendations for clinical practice in primary care [67] and is now widely used by practices, either on its own or combined with home or clinic monitoring, in management of the blood pressure of hypertensive patients. As a recommended procedure, this would not constitute an intervention additional to the routine management of hypertension, and should therefore be acceptable to participants, and it would also provide more robust and reliable outcome measurement [65,66]. Alternatively, practices are now required to record the blood pressure of all hypertensive patients within nine months; these data could potentially provide a baseline measure without going beyond normal practice routine.

Depression is less prevalent than hypertension in the United Kingdom adult population [5,7]. However, the lower rates of patients eligible from practice lists for the depression trial may reflect in part previous findings of inconsistent recording of depression on primary care databases [68]. The eligibility criterion for depression (PHQ-9 score 5 to 19 at recruitment) also led to a lower rate of cases from ‘screen eligible’ respondents in the depression condition (41.4%) than in the hypertension condition, where no additional baseline check was made (81.4%). Greater numbers of eligible patients might have been identified and recruited in the present study through alternative means such as prospective screening a general population of primary care patients for depression rather than working from past records of depression or low mood in practice databases. However, many patients excluded for a baseline score below 5 described fluctuating symptoms that a one-off PHQ-9 score might not capture [69]. This is consonant with findings from a recent population-based study screening repeatedly for major depression with PHQ-9 which observed fluctuations in reported symptoms and the predictive value of PHQ-9 was much lower in an intermediate range of the scale (score 7 to 14) than in higher and lower ranges [70]. Improvements between assessment and intervention in both depression and alcohol consumption outcomes have also been observed in a recent trial of brief intervention with depressed drinkers [71]. Repeated administration of PHQ-9 has been recommended for clinical practice [72] and been found to be appreciated by patients with mental health or substance use conditions [71,73], and this strategy might be preferable to determine eligibility and outcome in a trial around comorbid mild or moderate depression and excessive drinking. Patten and Schopflocher 72] recommend serial testing strategies for use with the general population, particularly in the immediate range of PHQ-9. Their strategy of six screenings at two-week intervals might represent a considerable increase in burden for research, however, four screenings over the same period might be feasible and provide more useful information than a single screen.

## Conclusions

A pilot study is now considered ‘an almost essential prerequisite’ to a full-scale study to ensure it will be successful [40]. Our findings indicate that a definitive trial in primary care of BI to reduce excessive alcohol consumption in hypertensive patients is logistically feasible and acceptable to practitioners (and, on face value, to patients) and should be carried out following the design of this pilot study but adopting a more robust aggregate outcome measure of blood pressure. Our sample size calculation suggests that 48 practices would need to be recruited for this, which would be achievable in a national trial. In addition, there has been a trend towards fewer and larger GP practices in England in recent years [55], suggesting that even fewer practices may ultimately suffice. Somewhat low rates of compliance among GP practices were encountered, particularly among non-research practices, with GPs citing lack of time or interest. It may be important to the successful recruitment of practices in a future trial to ensure that the study is fully discussed by all practice members rather than one partner or the practice manager ‘signing up’ the practice to a study without engaging their whole team.

A future trial would therefore be facilitated by measures such as ensuring practice participation costs are covered and that practices are incentivized by funding structures to participate in research, and engaging with local service provider networks. A grant funded through a national peer-reviewed funding stream would provide additional resource through research networks to engage more practices, overcoming some of these issues. Since database searches were not possible on all data management systems, it may be advisable for a future trial to consider limiting recruitment to those practices with systems and staff that can support such a search. This would potentially compromise external validity or generalizability, however, the considerable majority of practices agreeing to carry out the search strategy for this study were able to complete it. Although it was anticipated that excessive drinkers suffering from depression could also benefit from brief alcohol intervention to reduce their drinking, study design for such a trial may need further refinement to identify a broader strategy for screening, as well as an alternative approach to a single administration of PHQ-9 for assessing outcome, such as repeated administration or an alternative instrument (for example, the widely used Hospital Anxiety and Depression questionnaire) [74]. Qualitative research to explore patient experience of screening and assessment for depression would help inform this process [71]. Engagement with research appointments might also be improved in a trial of BI for excessive drinkers with depression through the use of text reminders or home visits. One hour of training, which would be sufficient for staff to deliver BI, would be most cost-effective if carried out within existing surgery meetings [75,76]. In respect of both hypertension and depression trials, identification of patients with these comorbidities would be facilitated with more widespread and regular alcohol screening by practices of their adult patients using validated tools, and incentivizing practices in this activity through national remuneration structures would be of benefit to this research.

Definitive trials on this basis will provide robust evidence for preventive approaches to healthcare provision that can improve the health of substantial patient populations and ease the burden on health services arising from their continued excessive drinking.

## Abbreviations

AUDIT: Alcohol Use Disorders Identification Tool; CONSORT: CONsolidated Standards Of Reporting Trials; DC: depression control; DI: depression intervention; FAST: Fast Alcohol Screening Test; GP: General Practitioner; HC: hypertension control; HI: hypertension intervention; NHS: National Health Service; PCT: Primary Care Trust; PHQ-9: 9-item Patient Health Questionnaire; SASQ: Single Alcohol Screening Question; (S)BI: (Screening and) Brief Intervention.

## Competing interests

The authors declare that they have no competing interests.

## Authors’ contributions

All authors have made an intellectual contribution to this research trial. As study investigators, EFSK, DNB, SH, PC, DT, EM and AC were responsible for identifying the research questions, designing the study, securing funding, and overseeing its implementation. GW was project manager for the study. RM had responsibility for intervention development and training. GBW, CW and RM conducted the study and delivered all interventions. DH advised on statistical considerations. CS advised on governance and trial procedures. GW led the drafting of this manuscript with CW. All authors had input into this process, commenting on each draft, and have read and approved the final version of the manuscript.

## Supplementary Material

Additional file 1 Search strategy and read codes used, including variations for different systems. Click here for file

Additional file 2 Brief advice sheets for hypertension and depression trials. Click here for file
