# Supplementary material for: Intervention to reduce excessive alcohol consumption and improve comorbidity outcomes in hypertensive or depressed primary care patients: two parallel cluster randomized feasibility trials
Source: Trials. 2014 Jun 19;15:235. doi: 10.1186/1745-6215-15-235 (PMC4076249; doi:10.1186/1745-6215-15-235)
Supplement: Additional file 1 — Search strategy and read codes used, including variations for different systems. [file 1745-6215-15-235-S1.docx]

**Additional File 1 –** Search strategy for practice databases

| **Search no.** | **‘SHARED’ READ CODES** | **‘EXCLUDED’ READ CODES** | **‘EITHER/OR’ (‘INCLUDED’) READ CODES** | **Results –**  **male patients** | **Results – female patients** |
| --- | --- | --- | --- | --- | --- |
| 1 | - **Current patient** - **Age** range 18-120 |  |  |  |  |
| ***Alcohol*** | | | | | |
| 2 | - **Current patient** - **Age** range 18-120 - **136** Weekly alcohol units range 0-120 |  |  |  |  |
| 3 | - **136** Weekly alcohol units range 22-120 - **Male patient** |  |  |  | **MALE PATIENTS ONLY** |
| 4 | - **136** Weekly alcohol units range 15-120 - **Female patient** |  |  | **FEMALE PATIENTS ONLY** |  |
| 5 | - **Current patient** - **Age** range 18-120 |  | - **388u** FAST range 0 to 16 - **38D2** SASQ any coding - **38D4** AUDIT-C range 0 to 12 |  |  |
| 6 | - **Current patient** - **Age** range 18-120 |  | - **388u** FAST range 3 to 16 - **38D2** SASQ positive - **38D4** AUDIT-C range 5 to 12 |  |  |
| 7 | - **Current patient** - **Age** range 18-120 - **38D3** AUDIT score range 0-40 |  |  |  |  |
| 8 | - **Current patient** - **Age** range 18-120 - **38D3** AUDIT score range 8-40 |  |  |  |  |

| ***Hypertension & alcohol*** | | | | | |
| --- | --- | --- | --- | --- | --- |
| 9 | - **Current patient** - **Age** range 18-120 |  | - **Systolic Blood Pressure** range 141-330 - **Diastolic Blood Pressure** range 91-220 |  |  |
| 10 | - **Current patient** - **Age** range 18-120 - **G2** Hypertensive disease |  |  |  |  |
| 11 | - **Current patient** - **Age** range 18-120 - **G2** Hypertensive disease - **136** Weekly alcohol units range 22-120 |  |  |  | **MALE PATIENTS ONLY** |
| 12 | - **Current patient** - **Age** range 18-120 - **G2** Hypertensive disease - **136** Weekly alcohol units range 15-120 |  |  | **FEMALE PATIENTS ONLY** |  |

| ***Depression & alcohol*** | | | | | |
| --- | --- | --- | --- | --- | --- |
| 13 | - **Current patient** - **Age** range 18-120 |  | - **388f** PHQ-9 range 0 to 27 - **388P** HADS depression inventory range 0 to 21 - Beck DI range 63-0 |  |  |
| 14 | - **Current patient** - **Age** range 18-120 |  | - **388f** PHQ-9 range 5 to 19 - **388P** HADS depression inventory range 8 to 21 - Beck DI range 30-14 |  |  |
| 15 | - **Current patient** - **Age** range 18-120 | - **8H49** referral to psychiatry - **8H4d** referral to psychogeriatrician | - **388f** PHQ-9 range 5 to 19 - **388P** HADS DI range 8 to 21 - Beck DI range 30-14 - **E2b** depressive disorder - **E2003** anxiety & depression |  |  |
| 16 | - **Current patient** - **Age** range 18-120 | - **8H49** referral to psychiatry - **8H4d** referral to psychogeriatrician | - **388f** PHQ-9 range 5 to 19 - **388P** HADS DI range 8 to 21 - Beck DI range 30-14 - **E2b** depressive disorder - **E2003** anxiety & depression - **1BT** Depressed Mood - **1BT-1** Low Mood |  |  |

| 17 | - **Current patient** - **Age** range 18-120 - **136** Weekly alcohol units range 22-120 - **Male patient** | - **8H49** referral to psychiatry - **8H4d** referral to psychogeriatrician | - **388f** PHQ-9 range 5 to 19 - **388P** HADS DI range 8 to 21 - Beck DI range 30-14 - **E2b** depressive disorder - **E2003** anxiety & depression - **1BT** Depressed Mood - **1BT-1** Low Mood |  | **MALE PATIENTS ONLY** |
| --- | --- | --- | --- | --- | --- |
| 18 | - **Current patient** - **Age** range 18-120 - **136** Wkly alc units range 15-120 - **Female patient** | - **8H49** referral to psychiatry - **8H4d** referral to psychogeriatrician | - **388f** PHQ-9 range 5 to 19 - **388P** HADS DI range 8 to 21 - Beck DI range 30-14 - **E2b** depressive disorder - **E2003** anxiety & depression - **1BT** Depressed Mood - **1BT-1** Low Mood | **FEMALE PATIENTS ONLY** |  |
| 19 | - **Current patient** - **Age** range 18-120 - **136** Wkly alc units range 22-120 - **Male patient** | - **8H49** referral to psychiatry - **8H4d** referral to psychogeriatrician | - **388f** PHQ-9 range 5 to 19 - **388P** HADS DI range 8 to 21 - Beck DI range 30-14 - **E2b** depressive disorder - **E2003** anxiety & depression - **1BT** Depressed Mood - **1BT-1** Low Mood |  | **MALE PATIENTS ONLY** |
| 20 | - **Current patient** - **Age** range 18-120 - **136** Wkly alc units range 15-120 - **Female patient** | - **8H49** referral to psychiatry - **8H4d** referral to psychogeriatrician | - **388f** PHQ-9 range 5 to 19 - **E2b** depressive disorder - **E2003** anxiety & depression - **1BT** Depressed Mood - **1BT-1** Low Mood | **FEMALE PATIENTS ONLY** |  |

Notes:

Most of the 25 GP practices who completed database searches used the EMIS data management system (LV and PCS versions); other programmes in use included SystmOne and Synergy. Numbers used to identify codes are based on the standard system of Read codes.

Very few adult patients had a record of screening for depression or alcohol use using formal validated tools. However, larger numbers of patients had diagnoses of depression or low mood entered; and units of alcohol consumed per week (code 136) had been recorded for most adult patients at some point since registering.

No date ranges were specified, except at the last five practices completing the searches where searches 19 and 20 were limited to past year only. This limit was applied to discover if this might reduce the number of potential cases screening positively for excessive drinking in the postal survey, but negatively for depression at the baseline appointment.
