# Supplementary material for: Intervention to reduce excessive alcohol consumption and improve comorbidity outcomes in hypertensive or depressed primary care patients: two parallel cluster randomized feasibility trials
Source: Trials. 2014 Jun 19;15:235. doi: 10.1186/1745-6215-15-235 (PMC4076249; doi:10.1186/1745-6215-15-235)
Supplement: Additional file 2 — Brief advice sheets for hypertension and depression trials. [file 1745-6215-15-235-S2.pdf]

Brief Advice

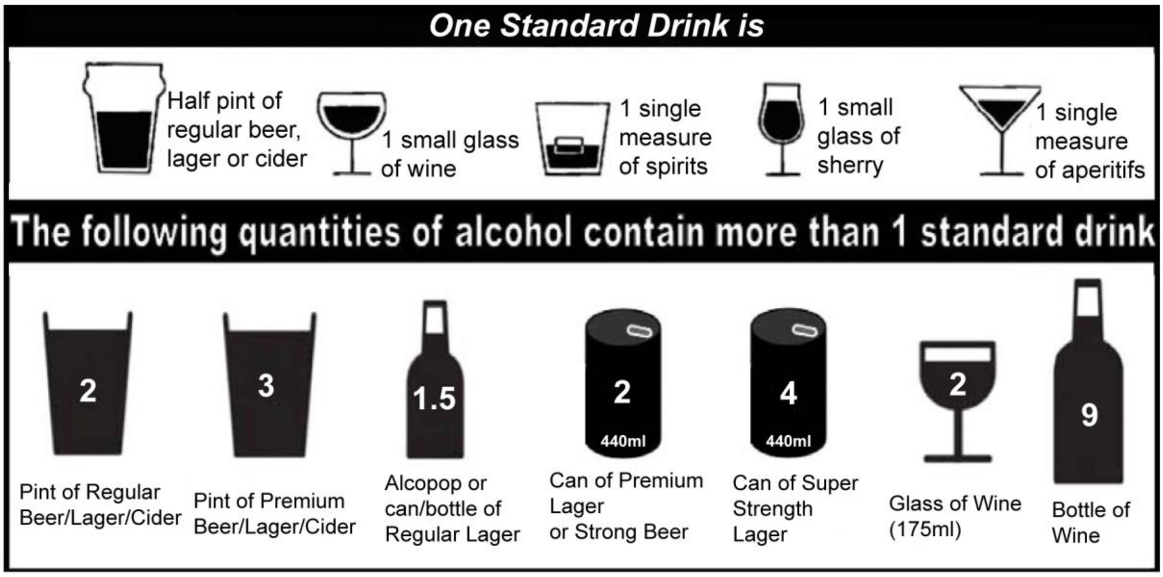

Are you at risk from drinking alcohol?

Risk	Men	Women	Common Effects
**SENSIBLE**	21 units or fewer per week or up to 4 units per day	14 units or fewer per week or up to 3 units per day	• Reduced risk of heart disease<br>• Sociability<br>• Increased relaxation
**HAZARDOUS/ HARMFUL**	22+ units per week or regular drinking of more than 4 units per day	15+ units per week or regular drinking of more than 3 units per day	• High blood pressure<br>• Increased risk of affecting current medication<br>• Less energy<br>• Risk of injury<br>• Reproductive problems<br>• Increased risk of liver disease<br>• Increased risk of cancer<br>• Possible alcohol dependence

- Binge drinking is considered to be drinking twice the daily limit in one sitting (8 units for men, 6 units for women).
- There are times when you will be at risk even after two or three drinks. For example, when exercising, operating heavy machinery, driving or if you are on certain medication.
- If you are pregnant, it is recommended that you completely abstain from drinking alcohol.
- As well as keeping to weekly and daily limits, it is recommended that 2 days of the week should be alcohol-free.

How do you feel?

Your screening score suggests you appear to be drinking at a rate that increases your risk of harm and you might be at risk of problems in the future. **What do you think?**

What is everyone else like?

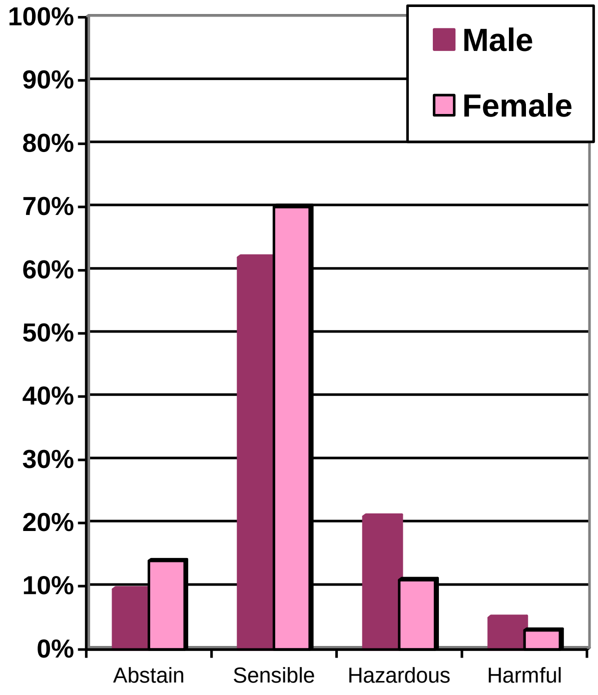

What are the benefits of cutting down?

Physical

- Reduced risk of high blood pressure
- Reduced risk of respiratory problems
- Reduced risk of strokes or heart attacks
- Reduced risk of injury / accidents
- Reduced risk of cancer
- Reduced risk of liver disease
- Lose weight
- Better physical shape

Psychological/Social/Financial

- Improved mood
- Improved relationships
- Reduced risks of drink driving
- Save money

Making your plan

- Plan activities and tasks at those times you usually drink
- When bored or stressed do something physically active instead of drinking
- Explore other hobbies or interests such as cinema, photography, painting, etc.
- Avoid going to the pub after work
- Have your first drink after starting to eat
- Quench your thirst with non-alcohol drinks before alcohol
- Avoid drinking in rounds or in large groups
- Switch to low alcohol beer/lager
- Take smaller sips when drinking alcohol
- Avoid or limit the time spent with “heavy” drinking friends

What targets should you aim for?

Men
4 or less standard drinks daily
21 or less standard drinks weekly

Women
3 or less standard drinks daily
14 or less standard drinks weekly
no drinks advised during pregnancy

Dependant Drinkers
Drinking alcohol is not advised. It is always important to seek support and further information from your GP before stopping drinking completely.

This brief intervention package is based on the How Much Is Too Much? Simple Structured Advice Intervention tool, developed by Newcastle University and the Drink Less materials originally developed at the University of Sydney as part of a W.H.O. collaborative study.

Newcastle University
Institute of Health&Society

NHS
NHS North of Tyne

Brief Advice

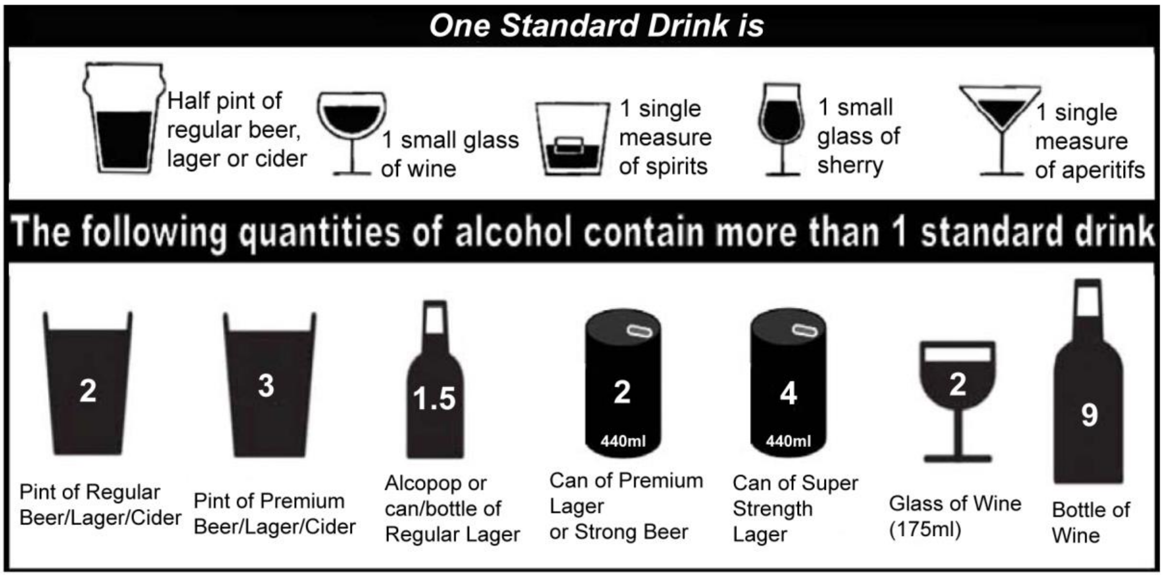

Are you at risk from drinking alcohol?

Risk	Men	Women	Common Effects
**SENSIBLE**	21 units or fewer per week or up to 4 units per day	14 units or fewer per week or up to 3 units per day	• Increased relaxation<br>• Sociability<br>• Reduced risk of heart disease
**HAZARDOUS/ HARMFUL**	22+ units per week or regular drinking of more than 4 units per day	15+ units per week or regular drinking of more than 3 units per day	• Depression/stress<br>• Insomnia<br>• Increased risk of affecting current medication<br>• Memory loss<br>• Less energy<br>• Impotence<br>• Reproductive problems<br>• Possible alcohol dependence

- Binge drinking is considered to be drinking twice the daily limit in one sitting (8 units for men, 6 units for women).
- There are times when you will be at risk even after two or three drinks. For example, when exercising, operating heavy machinery, driving or if you are on certain medication.
- If you are pregnant, it is recommended that you completely abstain from drinking alcohol.
- As well as keeping to weekly and daily limits, it is recommended that 2 days of the week should be alcohol-free.

How do you feel?

Your screening score suggests you appear to be drinking at a rate that increases your risk of harm and you might be at risk of problems in the future. **What do you think?**

What is everyone else like?

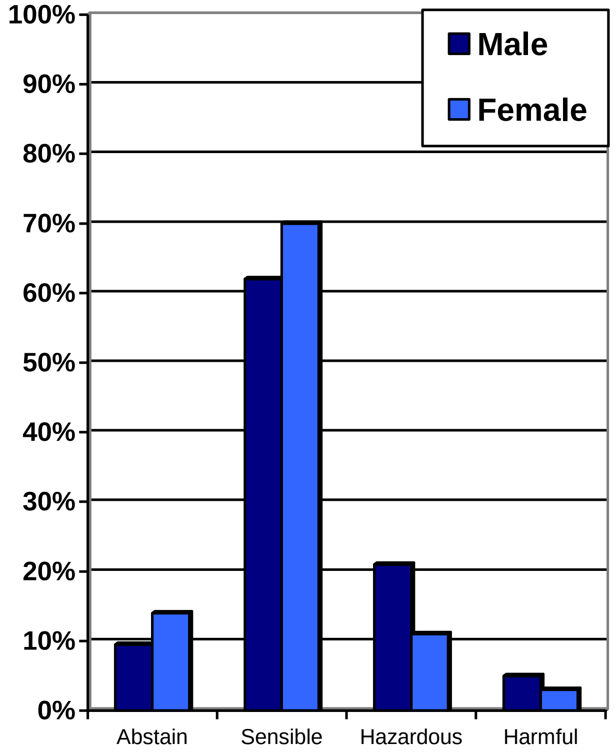

What are the benefits of cutting down?

Psychological/Social/Financial

- Improved mood
- Improved relationships
- Reduced risks of drink driving
- Save money
- No hangovers
- Improved memory

Physical

- Reduced risk of injury
- Reduced risk of high blood pressure
- Reduced risk of cancer
- Reduced risks of liver disease
- Reduced risks of brain damage
- Sleep better
- More energy
- Lose weight
- Better physical shape

Making your plan

- Plan activities and tasks at those times you usually drink
- When bored or stressed do something physically active instead of drinking
- Explore other hobbies or interests such as cinema, photography, painting, etc.
- Get involved in social activities such as walking clubs, book groups, theatre clubs, etc.
- Volunteer your time in the community
- Avoid going to the pub after work
- Have your first drink after starting to eat
- Quench your thirst with non-alcohol drinks before alcohol
- Avoid drinking in rounds or in large groups
- Switch to low alcohol beer/lager
- Take smaller sips when drinking alcohol
- Avoid or limit the time spent with “heavy” drinking friends

What targets should you aim for?

Men
4 or less standard drinks daily
21 or less standard drinks weekly

Women
3 or less standard drinks daily
14 or less standard drinks weekly
no drinks advised during pregnancy

Dependant Drinkers
Drinking alcohol is not advised. It is always important to seek support and further information from your GP before stopping drinking completely.

This brief intervention package is based on the How Much Is Too Much? Simple Structured Advice Intervention tool, developed by Newcastle University and the Drink Less materials originally developed at the University of Sydney as part of a W.H.O. collaborative study.

Newcastle University
Institute of Health&Society

NHS
NHS North of Tyne
